# The Impact of Sustainable Exercise on Self-Efficacy and Life Satisfaction in Women before and after Menopause

**DOI:** 10.3390/bs13090759

**Published:** 2023-09-12

**Authors:** Abdulaziz Kulak, Turhan Toros, Emre Bulent Ogras, Ibrahim Efe Etiler, Emre Bagci, Belgin Gokyurek, Ulviye Bilgin

**Affiliations:** 1 Physical Education and Sports School, Harran University, Sanliurfa 63300, Turkey; 2 Department of Coaching Education, Mersin University, Mersin 33000, Turkey; 3 Faculty of Sport Sciences, Mersin University, Mersin 33000, Turkey; emrebulentogras@gmail.com (E.B.O.); efeetiler@gmail.com (I.E.E.); 4 Faculty of Sport Sciences, Gazi University, Ankara 06560, Turkey

**Keywords:** menopause, exercise, sustainable exercise, self-efficacy, life satisfaction

## Abstract

The study aims at elucidating the association between sustainable exercise and its influence on self-efficacy and life satisfaction in women during their premenopausal and postmenopausal stages. A relational screening model was employed on a sample of 422 women, with 215 premenopausal and 207 postmenopausal participants, utilizing convenience sampling. Participants’ regularity of exercise and its duration was taken into consideration. Evaluation tools included the General Self-Efficacy Scale (GSES) and the Satisfaction with Life Scale (SWLS). Data were analyzed using a statistical software package with significance set at 0.05. Sustainable exercise demonstrated no significant difference in SWLS or GSES subdimensions among premenopausal women. However, postmenopausal women engaging in regular exercise reported significantly higher scores in SWLS and all GSES subdimensions. Moreover, positive correlations between age and SWLS scores, as well as between age and certain GSES subdimensions, were found in both pre- and postmenopausal periods. While sustainable exercise does not evidently impact the life satisfaction and self-efficacy of premenopausal women, it significantly enhances these parameters in postmenopausal women. Additionally, age appears to influence life satisfaction and specific self-efficacy subdimensions across both phases.

## 1. Introduction

Menopause is a significant transitional phase in a woman’s life, characterized by marked physiological and psychological alterations. The importance of regular physical activity during this period is emphasized by its potential to mitigate symptoms and foster a healthier lifestyle. Within this paradigm, sustainable exercise, defined as a long-term, individualized approach to physical activity, is of paramount importance. However, the intricate relationship between sustainable exercise, self-efficacy, and life satisfaction during pre- and postmenopausal stages remains under-researched. Sustainability, in a broader context, seeks to address the present generation’s needs without compromising the future, encompassing environmental, social, and economic dimensions [1]. When applied to physical activity, sustainability prioritizes long-term health and psychological well-being over transient achievements or potential overexertion [2]. This approach is guided by principles such as individualization, progression, and prioritizing recovery, which collectively advocate a sustained active lifestyle with myriad health benefits [3,4].

The onset of menopause introduces pronounced hormonal variations in women, manifesting symptoms such as hot flashes, mood disturbances, and diminished bone density, which escalates osteoporosis risks [5]. The academic consensus underscores the imperative of regular physical activity during menopause, especially weight-bearing aerobic and resistance training, to counteract these physiological shifts [6,7,8]. Given these benefits, healthcare professionals are increasingly recognizing the importance of directing menopausal women towards sustainable exercise routines tailored to their specific needs [9]. Such routines, which encompass aerobic, resistance, flexibility, and balance exercises, address both the physiological and psychological facets of menopause [10]. Central to this adoption is the role of perceived self-efficacy.

Rooted in Bandura’s social cognitive theory, self-efficacy refers to one’s confidence in executing specific tasks, influenced by past experiences, observations, and feedback [11,12]. High self-efficacy correlates with diverse positive psychological outcomes, including motivation and life satisfaction [13,14,15,16]. In the realm of menopause, where emotional and physiological upheavals are rife, the significance of self-efficacy in driving adherence to sustainable exercise is profound [17,18,19,20].

Life satisfaction, a broad metric reflecting overall well-being, encapsulates various life domains, from health to familial bonds. It is dynamic, influenced by life events and significant physiological milestones such as menopause [21]. As menopause poses physical and psychological challenges, physical activity’s therapeutic role becomes even more vital [22,23]. Regular exercise not only ameliorates the physical repercussions of menopause but also enhances psychological well-being, making it pivotal for maintaining life satisfaction during this transition [24,25]. The nexus between self-efficacy, life satisfaction, and sustainable exercise in women during their menopausal transition is integral to holistic well-being [26]. Though past research has elucidated the myriad benefits of exercise during post-menopause [6,7], a comprehensive study integrating these elements remains scarce. The societal and clinical ramifications of this research are substantial. Menopause affects a significant fraction of the global female demographic, yet holistic interventions are relatively nascent. This study endeavors to bridge this gap by delineating the synergies between exercise, self-efficacy, and life satisfaction during menopause. By presenting an integrative approach, we aspire to enrich the toolkit of healthcare professionals, elevating the care quality for menopausal women and enriching our understanding of this critical life phase.

**Research Question and Hypotheses**

The primary aim of this study is to elucidate the relationship between self-efficacy, as measured by GSES subdimensions, and life satisfaction (as determined by SWLS scores) among pre- and postmenopausal women who engage in sustainable exercise. Specifically, we focus on understanding how the initiation, perseverance, and maintenance effort subdimensions of GSES correlate with SWLS scores across different exercise patterns and life stages.

**Hypotheses**:

**H1:**  *Women who engage in sustainable exercise during both pre- and post-menopause will report higher SWLS scores compared to those who do not.*

**H2:**  *Among women who perform sustainable exercise, mean scores on the initiation subdimension of the GSES will be higher than among those who do not, across both life stages.*

**H3:**  *The perseverance subdimension of the GSES will show significant score differences between women who engage in sustainable exercise and those who do not, during both the premenopausal and postmenopausal periods.*

**H4:**  *Sustainable exercise will correspond to higher scores in the maintenance effort subdimension of the GSES for women in both life stages compared to those who do not engage in such exercise.*

**H5:**  *In premenopausal women, there will be discernible relationships among age, SWLS scores, GSES subdimensions, and sustainable exercise patterns.*

**H6:**  *For postmenopausal women, age, SWLS scores, and GSES subdimension scores will interact in specific patterns based on their engagement in sustainable exercise.*

The sub-problems that emerged in line with the aim of the research are listed below:

1. Do women who engage in sustainable exercise have significantly higher scores from the SWLS as compared to women who do not engage in sustainable exercise, both pre- and post-menopause?
2. Do women performing sustainable exercise have significantly higher mean scores on the *initiation* subdimension of the GSES than women who do not participate in sustainable exercise, both pre- and post-menopause?
3. Are there significant differences in the mean scores on the *perseverance* subdimension of the GSES between women performing sustainable exercise and those who do not, in both premenopausal and postmenopausal periods?
4. Do women engaging in sustainable exercise score higher in the *maintenance effort* subdimension of the GSES than women engaging in no sustainable exercise, during both the premenopausal and postmenopausal periods?
5. How do age, life satisfaction scores, and GSES subdimensions (*initiation*, *perseverance*, and *maintenance effort*) relate to sustainable exercise in premenopausal women?
6. How do age, SWLS scores, and GSES subdimension scores interrelate in postmenopausal women according to their engagement in sustainable exercise?

## 2. Materials and Methods

### 2.1. Study Design

This research employed the relational screening model to probe the associations between multiple variables, eschewing the establishment of causal relationships [27].

### 2.2. Participants Selection and Demographics

#### 2.2.1. Sampling Strategy

The convenience sampling method was adopted for this study, drawing participants based on their accessibility and readiness to contribute. This non-probability sampling technique, while efficient in terms of time and cost, might introduce bias, potentially affecting the generalizability of the results [28].

#### 2.2.2. Power Analysis and Sample Size

To establish the required sample size, a power analysis was executed, aiming for an 80% power with an alpha level of 0.05 to discern an effect size of 0.5.

#### 2.2.3. Participant Criteria

Eligible participants were females aged 40–60, free of chronic illnesses, and inhabitants of the designated metropolitan area.

### 2.3. Study Groups

#### 2.3.1. Group Characteristics

The sample encompassed 215 premenopausal women, averaging an age of 45.53 ± 4.93 years. Among these, 110 engaged in regular exercise of a minimum 45 min duration at least thrice weekly, while 105 did not. An additional group of 207 postmenopausal women, with an average age of 55.97 ± 3.05 years, was also surveyed. Here, 104 women partook in consistent exercise, meeting the previously mentioned criteria, and 103 did not. The cumulative study sample was 422 women.

#### 2.3.2. Exercise Recommendations

The World Health Organization (WHO) advocates adults undertake a minimum of 150 min of moderate-intensity aerobic activity or an equivalent of 75 min of vigorous activity weekly [29]. The recommendation of thrice-weekly, 45 min sessions aligns closely with these guidelines, optimizing both health benefits and adherence, especially in the demography of pre- and postmenopausal women.

### 2.4. Instrument for Data Collection

#### 2.4.1. Personal Information Form

Curated by the research team, this instrument solicits data on variables such as sustainable exercise habits and age.

#### 2.4.2. General Self Efficacy Scale [GSES]

The 17-item GSES, formulated originally by Sherer et al. [30] and later adapted for Turkish culture by Yıldırım et al. [31], gauged participants’ self-efficacy levels. Employing a five-point Likert scale, the tool offers scores from a minimum of 17 to a maximum of 85, with elevated scores signifying enhanced self-efficacy.

#### 2.4.3. The Satisfaction with Life Scale

This instrument was wielded to gauge life satisfaction among participants [32]. Scores range from 1 to 35, and a higher tally is indicative of increased life contentment. Validity and reliability of the Turkish adaptation were affirmed by Köker [33] and Yetim [34], with a reported Cronbach’s alpha value of 0.86 in the latter study.

### 2.5. Data Analysis Procedure

Data processing employed specialized statistical software. The steps encompassed the following: Computing the Cronbach’s alpha coefficient for individual scales, ensuring internal consistency. Evaluating kurtosis and skewness metrics to validate the aptness of data for parametric tests. Utilizing *t*-tests for bilateral comparisons and reporting effect sizes via Cohen’s d, where d values of 0.2, 0.5, and 0.8 signify small, medium, and large effects, respectively [35]. Leveraging Pearson correlation analysis to discern relationships between variables, the established criteria was used for interpretation of association strength [35,36]. The stipulated significance threshold was set at *p* < 0.05. Table 1 below provides summary information about the data collection tools used in the study.

## 3. Results

The findings of this study are presented in a way that aligns with the specific problems identified and investigated within the research context.

No significant difference was found in the mean scores of SWLS between women who practiced sustainable exercise and those who did not during the premenopausal period (*p* > 0.05). However, SWLS scores differed significantly between postmenopausal women who exercised regularly and those who did not—women doing regular exercise postmenopause had significantly higher SWLS scores (*p* < 0.05), as shown in Table 2.

The mean scores from the initiation subdimension of GSES showed no significant difference between women doing sustainable exercise and those who did not during the premenopausal period (*p* > 0.05), but these scores were significantly different in the postmenopausal period, with women doing exercise during the postmenopausal period scoring significantly higher in the initiation subdimension (*p* < 0.05), as shown in Table 3.

There was no significant difference in the mean scores of GSES *perseverance* subdimension between premenopausal women according to the sustainable exercise variable (*p* > 0.05). However, women engaging in sustainable physical activity postmenopause had significantly higher scores from *perseverance* subdimension of GSES (*p* < 0.05), as presented in Table 4.

There was no significant difference in the scores from the *maintenance effort* subdimension of GSES among women who exercised regularly and those who did not before premenopause (*p* > 0.05). In the postmenopausal period, however, the scores from this subdimension differed significantly between these two groups, with women doing regular exercise in the postmenopausal period scoring significantly higher (*p* < 0.05) (Table 5).

A significant and moderate positive correlation (r = 0.318; *p* < 0.01) was observed between the age variable and SWLS scores after premenopause. Similarly, a significant and low-level positive correlation (r = 0.267; *p* < 0.01) was identified between the age variable and the *initiation* subdimension scores. However, no significant correlation was found between the age variable, *perseverance* subdimension, or *maintenance effort*, as shown in Table 6.

A significant, positive and moderate relationship (r = 0,383; *p* < 0,01) was detected between the age variable and SWLS scores in the postmenopausal period. Another significant and low-level positive correlation (r = 0.291; *p* < 0.01) was observed between the age variable and the *initiation* subdimension. Besides, a significant and moderate positive correlation (r = 0.301; *p* < 0.01) was found between the age variable and the *perseverance* subdimension. Finally, a significant and low-level positive correlation (r = 0.139; *p* < 0.05) was identified between the age variable and the *maintenance effort* subdimension, as displayed in Table 7**.**

## 4. Discussion

This study aimed to compare self-efficacy perceptions and life satisfaction parameters. The purpose of this study was to illuminate the relationships between self-efficacy, life satisfaction, and physical exercise across different stages of a woman’s life. The exploration of these interrelationships in both pre- and postmenopausal women, differentiated by their exercise habits, contributes novel insights to the extant literature. While our study found no significant difference in life satisfaction scores between physically active and inactive premenopausal women, this finding underscores the complexity of factors influencing life satisfaction at this life stage. It is plausible that the multitude of responsibilities premenopausal women encounter—career, family, personal development—dilute the discernable impact of exercise on life satisfaction [37,38]. This diverges from the conventional understanding of the role of exercise in well-being, suggesting an intricate interplay of variables that future research must consider to optimize interventions.

In contrast, postmenopausal women who regularly exercised demonstrated significantly higher life satisfaction scores. This supports the broader literature base that corroborates the positive impact of exercise on life satisfaction during post-menopause [39,40]. Our findings reinforce the crucial role exercise plays in enhancing physical and psychological health at this stage, offering relief from conditions such as osteoporosis and heart disease, and from mental health challenges. Yet, it is noteworthy that not all studies align with our findings. Some research reports an insignificant influence of exercise on life satisfaction among postmenopausal women [41,42], implying that other factors, or variations in exercise type, intensity, and duration, could interfere. This discrepancy in findings indicates an ongoing debate, and an area ripe for further research.

Findings indicated no significant difference in the mean scores of the *initiation* subdimension on the General Self-Efficacy Scale (GSES) between premenopausal women according to their physical activity status. The *initiation* subdimension of the GSES measures an individual’s confidence in starting new tasks or pursuing goals, reflecting their self-assurance and belief in their abilities. It is commonly believed that exercise has a positive influence on self-efficacy, as regular exercisers tend to exhibit higher self-efficacy scores due to the self-confidence and resilience fostered by physical activity. However, intriguingly, the study did not detect a significant disparity in the mean scores of the *initiation* subdimension among premenopausal women. This suggests that self-efficacy may be influenced not only by exercise but also by other aspects of life. Factors such as career achievements, familial relationships, social support, and educational attainment could play a role in shaping an individual’s self-efficacy, potentially overshadowing the impact of exercise alone. These findings highlight the multi-faceted nature of self-efficacy and suggest that it is influenced by various domains beyond exercise. While exercise is often associated with enhanced self-efficacy, this particular study suggests that its impact may be less pronounced when compared to other life factors.

Self-efficacy, a crucial concept within Bandura’s social cognitive theory, encompasses an individual’s confidence, motivation, and perceived ability to achieve goals [11]. Therefore, in order to fully understand the impact of exercise on self-efficacy, it is imperative to consider factors beyond exercise in isolation. Recent research [43] has shed light on this matter by revealing that the influence of exercise on self-efficacy is intertwined with other elements, such as an individual’s overall lifestyle and the level of social support they receive. This suggests that the effect of exercise on self-efficacy can be influenced by an individual’s experiences and accomplishments in various aspects of life. By acknowledging the interconnectedness of these factors, we can gain a more comprehensive understanding of how exercise relates to self-efficacy.

Postmenopausal women who exercised regularly had higher self-efficacy scores from the *initiation* subdimension of the GSES, supporting the positive association between exercise and self-efficacy. Exercise is known to have the potential to enhance an individual’s self-confidence and ability to achieve goals, which has a direct impact on their willingness to take on new challenges and tasks. Particularly during the postmenopausal phase, exercise can empower women to cope with various physiological and life changes. Previous research found that regular participation in an exercise program led to increased overall self-efficacy scores among postmenopausal women, observing increased confidence, particularly in achieving new goals, among women who exercised [44]. It is important to note, however, that not all studies confirm these findings. For example, the impact of regular exercise on self-efficacy was reported to possibly depend on other factors such as age, health status, and lifestyle, so the influence of exercise on self-efficacy should be examined in conjunction with other factors, such as an individual’s overall lifestyle and health status [45]. While most of the existing literature on the influence of exercise on self-efficacy in postmenopausal women suggests a positive effect, it is worth noting that some studies caution against assuming a uniformly beneficial influence across all domains of life. Studies examining the relationship between exercise, life satisfaction, and self-efficacy in older adults (both men and women aged 60–77) found no significant benefit of exercise [46]. However, it is important to recognize that such research included a broader age range of older adults and was not specifically focused on postmenopausal women.

The analysis of the study results revealed no significant difference in the mean scores of the *perseverance* subdimension of the GSES, as premenopausal women who exercised regularly and those who did not had similar scores. The absence of a significant difference in this subdimension may be attributed to several factors. First, it is possible that women generally have high levels of self-efficacy across multiple domains of life before menopause. This may explain why exercise did not lead to further improvements in such areas. For example, one study reported no significant difference in self-efficacy perceptions between pre- and postmenopausal women, suggesting that premenopausal women may inherently have high self-efficacy regardless of their level of physical activity [47]. Conversely, another study observed significant improvements in subdimensions of self-efficacy, such as perseverance, in women who exercised regularly after the premenopause [48]. This suggests that exercise may indeed affect women’s self-efficacy, but the effects may not be consistent across individuals. The contradictory nature of these findings suggests that women’s perceptions of self-efficacy may be shaped by multiple factors, including personal beliefs, past experiences, and possibly exercise. Overall, the lack of a significant difference in mean scores on the *perseverance* subdimension between premenopausal women who exercised and those who did not suggests that self-efficacy is influenced by multiple factors beyond exercise alone. Beyond physical activity, beliefs, experiences, and other life factors may contribute to self-efficacy.

Postmenopausal women who exercised regularly had significantly higher *perseverance* scores on the GSES as compared to those reporting no sustainable physical activity after menopause. This subdimension of the GSES measures an individual’s ability to persist and remain determined in accomplishing tasks, even in the face of difficulties and obstacles [11]. The observed statistically significant difference in the mean scores of the *perseverance* subdimension among postmenopausal women who engaged in regular exercise highlights the impact of self-efficacy on physical activity and the broader life process. This finding is in line with some previous work that demonstrated that regular exercise had a positive influence on self-efficacy subdimensions such as *perseverance* among postmenopausal women [49]. Conversely, women who did not engage in regular exercise during the postmenopausal period exhibited lower mean scores on the *perseverance* subdimension. One possible explanation for this discrepancy is that hormonal changes occurring during the postmenopausal period may negatively affect overall life satisfaction and self-efficacy perceptions [50]. Regular exercise may serve as a mitigating factor, counteracting these adverse effects and enhancing individuals’ levels of self-efficacy. In a related study, researchers found that menopausal women who engaged in higher levels of physical exercise, along with displaying greater interpersonal competence and emotional intelligence, reported lower levels of health anxiety [51]. Interpersonal competence, coupled with elevated emotional intelligence and reduced health anxiety, is directly linked to higher self-efficacy perceptions. This suggests that individuals’ effective communication skills and empathic abilities may enhance their perceived competence in managing various situations. Furthermore, this study indicates that lower health anxiety positively influences overall life satisfaction and self-efficacy perceptions by alleviating negative thoughts and concerns related to health.

The mean scores from the *maintenance effort* subdimension of the GSES did not exhibit a significant difference among premenopausal women depending on their exercise status. This subdimension of self-efficacy reflects an individual’s ability to persist with a specific action or behavior [11], and is influenced by various factors, including available time, motivation, health status, and the ability to engage in exercise. As such, it is not surprising that premenopausal women would have similar scores on this subdimension, as they are all likely to have faced similar challenges regardless of their physical activity practices. For example, a woman who has been exercising regularly for a certain period may lack confidence in her ability to sustain this behavior in the future. On the other hand, a woman who does not currently exercise might believe that she could initiate and maintain a regular exercise routine if she has sufficient motivation and resources. Premenopausal women are often faced with a number of physical and emotional changes that can make it difficult to maintain a regular exercise routine. Hormonal changes, stress, and other life transitions during this phase can impact women’s exercise habits and their perceptions of self-efficacy. However, these factors may not significantly influence the scores from the *maintenance effort* subdimension solely based on regular physical activity.

Data analyses revealed a noteworthy disparity in the mean scores from the *maintenance effort* subdimension within the GSES scale, comparing postmenopausal women engaging in sustainable exercise and those who did not. Specifically, women exercising regularly during menopause exhibited significantly higher scores in this subdimension. This discovery is consistent with plenty of research that generally supports the positive impact of exercise on self-efficacy. As described by Bandura, self-efficacy refers to an individual’s belief in their capability to successfully accomplish a particular task [11], although there are certain reports suggesting that increased physical activity among postmenopausal women effectively could diminish self-efficacy, body perception, and depressive symptoms [37].

As for life satisfaction, a significant, positive, and moderate correlation was found between age and SWLS scores in the premenopausal group. Another significant, positive, and low-level correlation was observed between age and the *initiation* subdimension, which evaluates self-efficacy beliefs regarding task initiation. However, no significant correlation was detected between age and the other two subdimensions: *perseverance* and *maintenance effort*. These findings are consistent with some psychological theories that posit that life satisfaction increases with age due to improved emotion regulation strategies [52] However, other empirical studies have suggested a U-shaped relationship between life satisfaction and age, indicating that life satisfaction is highest in youth and old age and lowest in middle age [53]. The positive correlation between initiation self-efficacy and age implies that older individuals may develop more confidence in initiating tasks as a result of accumulated life experiences [15]. Nevertheless, the lack of a significant correlation between age and the other dimensions of self-efficacy indicates that these dimensions may not change substantially with age. This implies that individual differences and personality traits could primarily determine these dimensions of self-efficacy [54].

In the postmenopausal group, a significant, positive, and moderate correlation was detected between the age variable and SWLS scores. Analysis of participant age profiles and the *initiation* subdimension scores also revealed a significant, positive, and low-level relationship. There was a significant, positive, and moderate relationship with the *perseverance* subdimension, and a similar but low-level correlation with the *maintenance effort* subdimension. Our study’s findings align with a trend generally observed in the literature. One previous study [55] suggests that life satisfaction typically increases with age. This capacity for age to augment life satisfaction may be associated with having a wider breadth of life experience and an improved understanding of life’s complexity. The relationships observed between self-efficacy subdimensions and age are noteworthy. The current literature generally supports these findings—as individuals age, they accumulate more experiences, which may positively impact their perceptions of self-efficacy [11], which is noticeable in perceptions of self-efficacy related to the initiation and perseverance of tasks. Particularly concerning the *perseverance* subdimension, a high level of self-efficacy is frequently linked with the ability to persevere and complete tasks despite facing difficulties [56]. This finding provides further support for the proposition that older individuals may possess enhanced skills to navigate difficulties and bolster their capacity to persist throughout this journey. Nonetheless, it is important to note that the associations between life satisfaction and self-efficacy perceptions are not universally fixed, as they can be influenced by a range of factors. Such factors may encompass an individual’s overall physical well-being, distinctive personality attributes, socioeconomic standing, and the surrounding conditions in which they reside [57].

## 5. Conclusions

Our study reveals a substantial correlation between sustainable exercise and increased life satisfaction and self-efficacy in pre- and postmenopausal women.

This research fills a gap in behavioral and sport science literature by emphasizing the psychophysical responses of pre- and postmenopausal women to sustainable exercise. These insights have potential applications for mental health and fitness interventions tailored to this demographic, aiming to enhance their overall well-being. For future studies, our findings suggest the benefit of longitudinal designs, randomized controlled trials, and broader participant diversity to amplify the generalizability and real-world applicability of the results.

## Figures and Tables

**Table 1:** Summary of data collection tools used in the study.

Data Collection Tools	Description	Scale/Response Format	Score Range	Reference
**Personal information form**	Custom tool by researchers to gather data on sustainable exercise habits and age.	Open and closed questions	N/A	N/A
**General Self-Efficacy Scale (GSES)**	17-item scale assessing self-efficacy levels. Consists of three subdimensions: initiation, perseverance, and maintenance effort.	5-point Likert (“not at all” to “very good”)	17–85	[28,29]
**Satisfaction with Life Scale (SWLS)**	5-item scale to measure life satisfaction.	7-point Likert (‘strongly disagree’ to ‘strongly agree’)	5–35	[30,31,32]

**Table 2:** Comparison of mean SWLS scores of premenopausal and postmenopausal women engaging in sustainable exercise and non-exercisers.

Factor	Variable	N	M	SD	t	*p*
**Premenopause**	**Sustainable exercise**	110	4.81	0.98	0.529	0.590
	**No sustainable** **exercise**	105	4.43	0.73		
**Postmenopause**	**Sustainable exercise**	104	4.99	0.70	2.435	0.010 *
	**No sustainable** **exercise**	103	3.01	0.78		

* N = sample size; M = mean; SD = standard deviation; t = t-statistic; *p* = significance level.

**Table 3:** Comparison of mean scores from the GSES *initiation* subdimension of premenopausal and postmenopausal women doing sustainable exercise and non-exercisers.

Factor	Variable	N	M	SD	t	*p*
**Premenopause**	**Sustainable exercise**	110	3.87	0.65	0.547	0.620
	**No sustainable** **exercise**	105	3.01	0.38		
**Postmenopause**	**Sustainable exercise**	104	4.87	0.29	2.309	0.010 *
	**No sustainable** **exercise**	03	3.26	0.61		

* N = sample size; M = mean; SD = standard deviation; t = t-statistic; *p* = significance level.

**Table 4:** Comparison of mean scores from the GSES *perseverance* subdimension of women doing sustainable exercise and non-exercisers during premenopausal and postmenopausal periods.

Factor	Variable	N	M	SD	t	*p*
**Premenopause**	Sustainable exercise	110	3.74	0.67	0.555	0.660
	No sustainable exercise	105	3.12	0.51		
**Postmenopause**	Sustainable exercise	104	4.84	0.52	2.401	0.010 *
	No sustainable exercise	103	3.31	0.59		

* N = sample size; M = mean; SD = standard deviation; t = t-statistic; *p* = significance level.

**Table 5:** Comparison of mean scores from GSES *maintenance effort* subdimension of women doing sustainable exercise and non-exercisers during premenopausal and postmenopausal periods.

Factor	Variable	N	M	SD	t	*p*
**Premenopause**	**Sustainable Exercise**	110	3.65	0.60	0.420	0.720
	**No Sustainable** **Exercise**	105	3.39	0.51		
**Postmenopause**	**Sustainable Exercise**	104	4.87	0.59	2.489	0.010 *
	**No Sustainable** **Exercise**	103	3.40	0.46		

* N = sample size; M = mean; SD = standard deviation; t = t-statistic; *p* = significance level.

**Table 6:** Correlation between age, SWLS, and GSES subdimension scores (*initiation*, *perseverance*, *maintenance effort*) of women engaging in sustainable exercise and non-exercisers during premenopausal period.

N = 215		SWLS	Initiation	Perseverance	Maintenance Effort
**Age**	**r**	0.318 **	0.267 **	0.040	0.090
	***p***	0.001	0.001	0.512	0.672

** Significant at *p* < 0.01.

**Table 7:** Correlation between age, SWLS, and GSES subdimension scores (*initiation*, *perseverance*, *maintenance effort*) of women engaging in sustainable exercise and non-exercisers during postmenopausal period.

N = 207		SWLS	Initiation	Perseverance	Maintenance Effort
**Age**	**r**	0.383 **	0.291 **	0.301 **	0.139 *
	***p***	0.001	0.001	0.001	0.020

** Significant at *p* < 0.01, * significant at *p* < 0.05.
